# Preoperative false-negative transthoracic echocardiographic results in native valve infective endocarditis patients: a retrospective study from 2001 to 2018

**DOI:** 10.1186/s12947-020-00229-8

**Published:** 2021-01-02

**Authors:** Zuning Ren, Jian Zhang, Hongjie Chen, Xichao Mo, Shaohang Cai, Jie Peng

**Affiliations:** Department of Infectious Diseases, Nanfang Hospital, Southern Medical University, Guangzhou, China

**Keywords:** Infective endocarditis, Echocardiogram, Echocardiography, False negative, Surgery, Retrospective study

## Abstract

**Background:**

Infective endocarditis (IE) is a lethal disease that is difficult to diagnosis early. Although echocardiography is one of the most widely used diagnostic technique, it has limited sensitivity. This study surveyed the clinical features of IE patients who underwent surgery and compared transthoracic echocardiography and histological findings to explore the factors related to false-negative echocardiographic results.

**Methods:**

Medical records were extracted from IE patients consecutively hospitalized between June 2001 and June 2018.

**Results:**

A total of 182 patients with native valve IE who underwent surgery were included. Compared to the non-surgery group, the surgery group was more likely to have pre-existing valvular lesions and more serious cardiac conditions and a relative lack of signs of infection and cerebrovascular events, leading to a lower proportion of “definite cases” before surgery. The false-negative rate of echocardiography was 14.5%. Echocardiography has significant disadvantages in diagnosing perivalvular abscesses, valve perforations, and left-sided endocarditis, especially for subjects with both aortic and mitral valve infections. The multivariate analysis identified congenital heart disease and small vegetations (< 10 mm) as independent predictors of false-negative echocardiography results. Conversely, fever and heart murmurs on admission served as protective factors.

**Conclusions:**

Under some circumstances, echocardiography provides inconsistent results compared with surgical findings, and negative echocardiography results do not rule out IE. The diagnosis of IE depends on comprehensive evaluations using multiple methods.

**Supplementary Information:**

The online version contains supplementary material available at 10.1186/s12947-020-00229-8.

## Background

Infective endocarditis (IE) is a lethal disease caused by various pathogens such as bacteria, fungi, and rickettsia that directly invade the cardiac valves or mural endocardium [1]. Despite significant technological advances in medical and surgical therapies, IE carries risks of high mortality and poor prognosis [2]. The early identification and diagnosis of this condition remain major challenges [3].

Echocardiography, either transthoracic echocardiography (TTE) or Transesophageal echocardiography (TOE), is the primary choice for the diagnosis of IE [4]. Valvular vegetations are the diagnostic and pathologic markers of IE and can be preliminarily screened out by echocardiography. In some cases, a clinical diagnosis of IE can be made in the absence of vegetations by using the modified Duke criteria [5]. The sensitivities for the diagnosis of vegetations in native valves are 70% for TTE and 96% for TOE [6, 7]. Their identification may be difficult in IE patients with pre-existing valvular lesions like mitral valve prolapse, degenerative cardiac valve disease, prosthetic valves, small vegetations, recent embolization and in vegetation-negative endocarditis. Therefore echocardiographic results must be interpreted with caution, synthesizing patient’s clinical features and their likelihood of IE.

The role of the pathologist is often decisive. Histological assessment of cardiac valves to demonstrate vegetations and valvular inflammation remains the gold standard for IE diagnosis [8], especially for complicated cases with atypical clinical manifestations and auxiliary examination results, when bacteriologists fail to isolate a microorganism [9]. Besides, histological analysis can distinguish blood culture-negative endocarditis from noninfective causes of endocarditis, particularly neoplastic or autoimmune disease [4]. However, due to the high cost of surgical biopsy and pathological examination, some cases are never histologically diagnosed.

We consecutively collected data from IE patients who underwent surgery at a comprehensive teaching hospital in southern China to provide better evidence-based medical evidence and identify factors related to false-negative TTE results.

## Methods

### Diagnostic criteria

The definition of IE was based on the 2015 European Society of Cardiology algorithm for diagnosis of infective endocarditis [4], which mainly includes the pathological diagnostic criteria and the modified Duke criteria.

Pathological examination served as the gold standard for IE diagnosis and had to meet at least one of the following criteria: microorganisms demonstrated by culture or histological examination of a vegetation; a vegetation that has embolized or an intracardiac abscess specimen; or the presence of pathological lesions, vegetation, or intracardiac abscesses by histological examination showing active endocarditis. Pathologists were blinded to clinical parameters and echocardiographic results when diagnosing vegetation samples [4].

The modified Duke criteria (adapted from Li et al. [5]) were used to clinically diagnose cases classified as either definite or suspected. Three echocardiographic findings are the major criteria in IE diagnosis: vegetation, abscess, or pseudoaneurysm and new dehiscence of a prosthetic valve [7]. The echocardiographic definitions are listed in the Additional file 1.

Surgery was performed during the course of the appropriate antimicrobial therapy and was indicated for at least one of the following conditions, which were in accordance with the current guidelines [4, 10]: severe valvular dysfunction in the presence of heart failure, abscess or perivalvular extension, large vegetations at high risk of embolization (or recurrent embolization during antibiotic treatment), and failure of conservative medical treatment.

### Study sample

We consecutively collected data from 313 consecutive IE cases through the electronic medical records system of Nanfang Hospital, a comprehensive teaching hospital, between June 2001 and June 2018. The partial results of this study were published in 2019 [11]. We excluded 11 cases of prosthetic valve IE and 2 cases with a history of pacemaker transplantation.

Data included demographic information, predisposing factors, clinical manifestations, echocardiography results, pathologic findings, and in-hospital mortality.

This clinical study was a retrospective and descriptive study performed in accordance with the principles of the Helsinki declaration.

### Statistical analyses

All analyses were performed using SPSS version 25.0 (IBM Corp., Armonk, NY, USA). Continuous variables with normal distributions are expressed as mean ± standard deviation; categorical variables are expressed as frequency and percentage. Paired χ2 tests (McNemar tests) were used to assess differences between echocardiogram and surgical histopathology results. Univariate comparisons were evaluated with χ2 tests or Fisher’s exact test for categorical variables, as appropriate. Variables with theoretical clinical importance and those that achieved a *P* < 0.10 in the univariate analysis were included in the binary logistic regression analysis. A forward conditional method was used to select the most useful predictors for inconsistency between echocardiographic and surgical findings. Results were considered statistically significant at the 0.05 level.

## Results

### Difference between the surgery and non-surgery groups

A total of 300 patients were consecutively diagnosed with native valvular endocarditis, and 182 underwent surgery. Tables 1 and 2 details the basic information, clinical features, echocardiographic findings, diagnostic basis, and in-hospital mortality of the surgery and non-surgery groups.

**Table 1:** Basic information, clinical features, diagnostic information and mortality of 310 IE patients

Variable	Total *N* = 300	Surgery *N* = 182	None surgery *N* = 118	*P*
Previous cardiovascular conditions or cardiac diseases	126 (42.0)	85 (46.7)	41 (34.5)	0.003
Degenerative calcific valvular disease	27 (9.0)	18 (9.9)	9 (7.6)	0.606
Rheumatic heart disease	59 (19.7)	41 (22.5)	18 (15.1)	0.122
Congenital heart disease	58 (19.3)	42 (23.1)	16 (13.4)	0.053
Clinical features				
Fever	251 (83.7)	141 (77.5)	110 (92.4)	0.000
Heart murmurs	253 (84.3)	162 (89.0)	91 (76.5)	0.006
Ischemic stroke	65 (21.7)	31 (17.0)	34 (28.6)	0.016
Hemorrhagic stroke	26 (8.7)	10 (5.5)	16 (13.4)	0.015
Heart insufficiency (NYHA II ~ IV)	175 (58.3)	124 (68.1)	51 (42.9)	0.000
^b^Acute congestive heart failure	64 (21.3)	41 (22.5)	23 (19.3)	0.531
Positive blood culture	172 (57.3)	93 (51.1)	79 (66.4)	0.007
*Staphylococcus aureus*	43 (14.3)	14 (7.7)	29 (24.4)	0.000
Modified Duke’s criteria				
Definite IE	166 (55.3)	81 (44.5)	85 (71.4)	0.000
Suspected IE	113 (37.7)	80 (44.0)	33 (27.7)	0.005
Excluded	–	^a^21 (11.5)	–	–
Pathological criteria				
Pathological confirmed	–	^a^173 (95.1)	–	–
Pathological excluded	–	^a^9 (4.9)	–	–
In-hospital death	32 (10.7)	8 (4.4)	24 (20.2)	0.000

^a^These data were only available to surgery group

^b^Patients developed acute pulmonary edema

*NYHA* New York Heart Association

**Table 2:** Echocardiographic results of 310 IE patients

Variable	Total *N =* 300	Surgery *N =* 182	None surgery *N =* 118	*P*
Positive echocardiographic results				
Vegetation	266 (88.7)	157 (86.3)	109 (91.6)	0.103
Left heart	193 (64.3)	117 (64.3)	76 (63.9)	0.983
Only on aortic valve	70 (23.3)	42 (23.1)	28 (23.5)	0.896
Only on mitral valve	101 (33.7)	57 (31.3)	44 (37.0)	0.285
Aortic valve and mitral valve	22 (7.3)	18 (9.9)	4 (3.4)	0.035
Right heart	58 (19.3)	32 (17.6)	26 (21.8)	0.340
Only on tricuspid valve	54 (18.0)	29 (15.9)	25 (21.0)	0.247
Only on pulmonic valve	4 (1.3)	3 (1.6)	1 (0.8)	0.940
Left heart and right heart	9 (3.0)	2 (1.1)	7 (5.9)	0.040
Perivalvular abscess	4 (1.3)	3 (1.6)	1 (0.8)	0.940
Aortic sinus aneurysm	6 (2.0)	4 (2.2)	2 (1.7)	1.000
Perforation	20 (6.7)	11 (6.0)	9 (7.6)	0.591
Severe valve insufficiency	176 (58.7)	122 (67.0)	54 (45.4)	0.000
Size of vegetations				
> 1 cm	236 (78.7)	144 (79.1)	92 (77.3)	0.811

The surgery group was more likely to suffer from previous cardiovascular conditions or cardiac diseases (85% vs. 41%, odds ratio [OR] = 0.488, 95% confidence interval [CI]: 0.301–0.786) and more likely to present as heart murmurs (89.0% vs. 76.5%, OR = 2.403, CI: 1.277–4.525) and heart insufficiency (New York Heart Association class II to IV) (68.1% vs. 42.9%, OR = 2.809, CI: 1.739–4.536) at admission. Conversely, evidence of infections like fever (77.5 vs. 92.4%, OR = 0.250, CI: 0.113–0.555) and positive blood culture results (51.1% vs. 66.4%, OR = 0.516, CI: 0.319–0.835), as well as cerebrovascular events like hemorrhagic stroke (5.5% vs. 13.4%, OR = 0.371, CI: 0.162–0.848) and ischemic stroke (17.0% vs. 28.6%, OR = 0.507, CI: 0.291–0.884), were significantly less common in the surgery group.

Patients in the surgery group were more likely to have both aortic and mitral valve infections (9.9% vs. 3.4%, OR = 3.128, CI: 1.031–9.486) and suffer from severe valve insufficiency (67.0% vs. 45.4%, OR = 2.410, CI: 1.497–3.879). However, the left and right heart valves were less likely to be simultaneously infected (1.1% vs. 5.9%, OR = 0.176, CI: 0.036–0.863).

In the surgery group, there were clinically fewer definite IE cases (44.5% vs. 71.4%, OR = 0.311, CI: 0.189–0.512), but more suspected IE cases (44.0% vs. 27.7%, OR = 2.020, CI: 1.229–3.322) before surgery. Nine cases did not meet the histological diagnostic criteria but had a high level of clinical evidence to support the diagnosis of IE. In-hospital mortality was significantly lower in the surgery group (4.4% vs. 20.2%, OR = 0.180, CI: 0.079–0.417).

### Pathological and echocardiographic results in the surgery group

The pathological and echocardiographic results of 182 surgery patients are shown in Table 3. The preoperative echocardiographic findings mainly included vegetations (86.3%), perivalvular abscess (1.6%), perforation (6.0%), and aortic sinus aneurysm (2.2%). Echocardiography identified less frequently the presence of perivalvular abscess (1.6% vs. 7.1%, *P* = 0.013) and valve perforation (6.0% vs. 13.7%, *P* = 0.013) compared to surgical findings as gold standard. Based on a comparative analysis, the location of vegetations was significantly different between echocardiography and surgical findings (86.3% vs. 95.1%, *P* = 0.007). Left-sided endocarditis was more likely to be missed by echocardiography (64.3% vs. 70.9%, *P* = 0.050), especially in patients with both aortic and mitral valve infections (9.9% vs. 14.3%, *P* = 0.039).

**Table 3:** McNemar tests results between pathological and transthoracic echocardiographic results

	Pathology	Echocardiography	McNemar test
			*P*
Perivalvular abscess	13 (7.1)	3 (1.6)	0.013
Perforation	25 (13.7)	11 (6.0)	0.013
Aortic sinus aneurysm	4 (2.2)	4 (2.2)	1.000
Vegetation	173 (95.1)	157 (86.3)	0.007
Left heart	129 (70.9)	117 (64.3)	0.050
Aortic valve	40 (22.0)	42 (23.1)	0.832
Mitral valve	63 (34.6)	57 (31.3)	0.180
Aortic valve and mitral valve	26 (14.3)	18 (9.9)	0.039
Right heart	33 (18.1)	32 (17.6)	1.000
Tricuspid valve	27 (14.8)	29 (15.9)	0.687
Pulmonic valve	6 (3.3)	3 (1.6)	0.250
Left heart and right heart	6 (3.3)	2 (1.1)	0.219
^a^Abnormal cardiac structure	12 (6.6)	8 (4.4)	0.388

^a^Including vegetations found on atrial septum, ventricular septum, ductus arteriosus, etc.

The majority (70.9%) of echocardiographic and surgical results were completely consistent. Negative echocardiographic results were observed in 25 (13.7%) cases. The remaining 28 cases (15.4%) showed misdiagnosis based on echocardiography (wrong distribution and quantity of valvular lesions) before surgery. The false-negative rate was 14.5% (25/173). We divided 310 IE patients into two time period groups (Group 2001–2009 and Group 2010–2018) based on the time of admission and compared the false-negative rate of echocardiography between two groups. We found no significant difference between two groups (10.9% vs 15.0, *P* = 0.466).

### Factors related to the false-negative TTE results

To investigate the specific factors that caused the false-negative results of echocardiographic findings compared to histological results, we performed univariate and multivariate analyses (Table 4). The multivariate analysis revealed that congenital heart disease (26.2% vs. 10.0%, OR = 2.907, 1.062–7.956) and small-size vegetations (< 10 mm; 37.5% vs. 8.7%, OR=8.197, CI:2.841-23.256) were independent predictors of false-negative results on echocardiography. Fever (10.6% vs. 24.4%, OR = 0.309, 0.108–0.882) and heart murmurs (11.1% vs. 35.0%, OR = 0.165, CI: 0.050–0.546) at admission served as protective factors.

**Table 4:** Factors associated with the false negative results of echocardiographic results

Variable	Category	Number	Inconsistence(%)	Univariate analysis				Multivariate analysis			
				***P***	OR	95%CI		***P***	OR	95%CI	
						lower	upper			lower	upper
**Previous cardiovascular conditions or cardiac diseases**											
Congenital heart disease	yes	42	11 (26.2)	0.008	3.194	1.322	7.715	0.038	2.907	1.062	7.956
	no	140	14 (10.0)								
Degenerative cardiac valve disease	yes	18	3 (16.7)	0.984	1.291	0.645	4.825				
	no	164	22 (13.4)								
Rheumatic heart disease	yes	41	9 (22.0)	0.083	2.197	0.890	5.428	0.080			
	no	141	16 (11.3)								
**Clinical features**											
Fever	yes	141	15 (10.6)	0.024	0.369	0.151	0.900	0.028	0.309	0.108	0.882
	no	41	10 (24.4)								
Heart murmurs	yes	162	18 (11.1)	0.010	0.232	0.082	0.658	0.003	0.165	0.050	0.546
	no	20	7 (35.0)								
Embolism	yes	40	8 (20.0)	0.193	1.838	0.728	4.639				
	no	142	17 (12.0)								
Positive blood culture results	yes	94	10 (10.6)	0.21	0.579	0.245	1.368				
	no	88	15 (17.0)								
**Size of vegetations**	< 1 cm	32	12 (37.5)	0.000	6.330	2.532	15.873	0.000	8.197	2.841	23.256
	> 1 cm	150	13 (8.7)								

## Discussion

IE is a fatal disease with high mortality despite novel diagnostic and therapeutic strategies. Timely and early diagnosis of IE remains a challenge. Our study was aimed to clarify the characteristics of IE patients who underwent a surgery over an 18-year period in our hospital and to identify factors related to the false-negative echocardiography results. To our knowledge, this is the largest, long-term study on IE performed in our region.

### Features of patients in the surgery group

For non-surgery patients, we adopted the modified Duke criteria for diagnosis, but only those who met the criteria of “definite IE” or “suspected IE” could be enrolled to ensure the reliability of the collected data. For patients in the surgery group, beyond the clinical diagnostic criteria, pathological results played a more critical role as the gold standard for diagnosis. A subset of patients had been never considered to have IE until intraoperative findings of vegetations or intracardiac abscesses. In our study, more patients in the surgical group showed severe valve insufficiency and heart insufficiency, which is also one of the indications for surgery. Due to the low positive rate of blood culture, atypical clinical symptoms or other reasons, a range of cases could not exactly match modified Duke criteria before the surgery until the histopathological result supported definite IE. Therefore, there were clinically fewer definite IE cases but more suspected IE cases before surgery. By comparing differences between echocardiographic and surgical findings within the surgery group, we found that missed diagnosis by echocardiography was more likely when perivalvular abscesses and valve perforation developed or when vegetations affected both the mitral and aortic valves. This is a novel finding; one possible explanation is that the pre-existing valvular disease with structural abnormalities and calcification are more likely to affect both the mitral and aortic valves, which may affect echocardiographic observations.

The International Collaboration on Endocarditis-Prospective Cohort Study reported that the average in-hospital mortality of IE was 18% worldwide [12]. In contrast, the in-hospital mortality of our study was 10.7%. The mortality of patients who underwent surgery was almost one-sixth of that of patients who did not undergo surgery in our study. Several previous studies pointed out that surgery was independently associated with a lower risk of in-hospital mortality [13–15]. We previously performed a multivariate analysis in 313 cases of IE (including prosthetic valve endocarditis) [11] and identified intravenous drug addiction, prosthetic valve endocarditis, hemorrhagic stroke, acute congestive heart failure, renal insufficiency, left-sided endocarditis, and early surgery as independent predictors of in-hospital mortality. According to this data, we concluded that the surgery and less frequent occurrence of hemorrhagic stroke were protective factors for good prognosis of IE in the surgery group. This finding highlights that surgery is a crucial treatment for improving prognosis.

### Factors related to the false-negative results of echocardiography

Our false-negative TTE rate was 14.5%, similar to other studies [7, 16]. Previous reports indicated that an echocardiographic diagnosis of endocarditis may be correct but sometimes incomplete [16, 17]. Regardless of the possible error in subjective assessments and operation caused by ultrasound technicians, the most common explanations for false-negative or erroneous echocardiographic results are atypical position of the vegetations, and small vegetations [6]. Our findings were in line with the previous conclusions.

Both TTE and TOE may produce false-negative results if vegetations are small or have embolized. Many embolic events occur during the first 2 weeks after initiation of antibiotic therapy. The key point is the beginning of antibiotic treatment before surgery. In this circumstances it is important to specify the management of antimicrobial therapy and order an echocardiography at early time.

The multivariate analytic results showed that congenital heart disease and vegetation size < 10 mm were risk factors for false-negative echocardiographic results, while fever and heart murmurs were protective factors. The latter two factors are typical manifestations of infective endocarditis and might cause alarm among clinicians, thus affecting the echocardiographic diagnosis.

Clinicians must be aware that echocardiography sensitivity is not 100%, and negative echocardiography results do not rule out IE. Sometimes echocardiography should be repeated several times [2]. Significant progress in echocardiography has taken place in the last decades transitioning from 2-dimensional (2D) imaging to the increasing role of 3-dimensional (3D) imaging modality. The real-time 3D TOE is recommended as it allows better characterization of IE vegetation [18].

Some studies have pointed out that the diagnostic sensitivity of TTE in *S. aureus-*related IE is significantly lower, while TEE significantly improves the diagnostic sensitivity [19]. However, another publication expressed reservations [20]. Our study also attempted to explore the effect of blood culture results on the accuracy of echocardiographic diagnosis of IE, but the results were not satisfactory. In our previous study [11], we mentioned that the blood culture positive rate of IE in our hospital was only 58.2% due to antibiotic abuse and other reasons, which was roughly consistent with the results of the present study. We speculated that this might affect the univariate analysis results.

Pathological examination of cardiac valves remains the gold standard for IE diagnosis. However, 9 cases of definite IE in our study did not meet the pathological diagnosis criteria. Detachment or disintegration of small vegetations after antibiotic therapy probably responsible for the false negative results of pathological results. In the absence of pathological evidence, the sensitivity of clinical diagnosis of IE using the modified Duke’s standard alone is ~ 80% [21]. Still, Duke’s standard is also an important reference when we fail to obtain ideal pathological results.

Whether in the surgery or non-surgery group, the sensitivity of TTE remains a question. Factors related to false-negative echocardiographic results were also existed in non-surgical group. Therefore, the study was meaningful for both the surgical group and the non-surgical group.

Besides, the value of integrated diagnostic strategies using multimodality imaging is emerging. The multimodality imaging has assumed a pivotal role in the clinical decision making. As echocardiography has several limitations, the integration with other imaging modalities (computed tomography, magnetic resonance imaging, nuclear imaging) becomes often necessary.

This was a single-center study performed in a general teaching hospital, so the findings may not be applicable to all populations and areas. Besides, referral bias should be taken into consideration when describing the echocardiographic and surgical outcomes of IE, as patients with more complicated and serious illness were more likely to be treated at a tertiary hospital [22]. Finally, the echocardiographic and pathological results are somewhat subjective, making detailed comparisons difficult.

## Conclusion

Compared to the non-surgery group, the surgery group was more likely to have pre-existing valvular lesions and more serious cardiac conditions and fewer signs of infection and cerebrovascular events, leading to a lower proportion of “definite cases.” Missed diagnosis by echocardiography was more likely to occur when perivalvular abscess and valve perforation developed, and when vegetations affected the mitral and aortic valves. Congenital heart disease, fever, heart murmurs manifested at admission, and vegetations with small size (< 10 mm) were independent predictors of false-negative echocardiography results.

## Supplementary Information

**Additional file 1.** The anatomical and echocardiographic definitions.

## Data Availability

The datasets used and/or analyzed during the current study are available from the corresponding author on reasonable request.

## Abbreviations

IE: Infective endocarditis
TTE: Transthoracic echocardiography
TOE: Transesophageal echocardiography
OR: Odd ratio
CI: Confidence interval

## References

[1] Cahill TJ, Prendergast BD (2016). Infective endocarditis. Lancet..

[2] Thuny F, Grisoli D, Collart F, Habib G, Raoult D (2012). Management of infective endocarditis: challenges and perspectives. Lancet..

[3] Cahill TJ, Baddour LM, Habib G, Hoen B, Salaun E, Pettersson GB (2017). Challenges in infective endocarditis. J Am Coll Cardiol.

[4] Habib G, Lancellotti P, Antunes MJ, Bongiorni MG, Casalta JP, Del Zotti F, Dulgheru R, El Khoury G, Erba PA, Iung B, Miro JM, Mulder BJ, Plonska-Gosciniak E, Price S, Roos-Hesselink J, Snygg-Martin U, Thuny F, Tornos Mas P, Vilacosta I, Zamorano JL (2015). 2015 ESC guidelines for the management of infective endocarditis: the task force for the Management of Infective Endocarditis of the European Society of Cardiology (ESC). Endorsed by: European Association for Cardio-Thoracic Surgery (EACTS), the European Association of Nuclear Medicine (EANM). Eur Heart J.

[5] Li JS, Sexton DJ, Mick N, Nettles R, Fowler VG, Ryan T (2000). Proposed modifications to the Duke criteria for the diagnosis of infective endocarditis. Clin Infect Dis.

[6] Mügge A, Daniel WG, Frank G, Lichtlen PR (1989). Echocardiography in infective endocarditis: reassessment of prognostic implications of vegetation size determined by the transthoracic and the transesophageal approach. J Am Coll Cardiol.

[7] Habib G, Badano L, Tribouilloy C, Vilacosta I, Zamorano JL, Galderisi M (2010). Recommendations for the practice of echocardiography in infective endocarditis. Eur J Echocardiogr.

[8] Sanchez-Enrique C, Vilacosta I, Moreno HG, Delgado-Bolton R, Perez-Alonso P, Martinez A (2014). Infected marantic endocarditis with leukemoid reaction. Circulation J.

[9] Lepidi H, Durack D, Raoult D (2002). Diagnostic methods: current best practices and guidelines for histologic evaluation in infective endocarditis. Infect Dis Clin N Am.

[10] Baddour LM, Wilson WR, Bayer AS, Fowler VG, Tleyjeh IM, Rybak MJ (2015). Infective endocarditis in adults: diagnosis, antimicrobial therapy, and management of complications: a scientific statement for healthcare professionals from the American Heart Association. Circulation..

[11] Ren Z, Mo X, Chen H, Peng J (2019). A changing profile of infective endocarditis at a tertiary hospital in China: a retrospective study from 2001 to 2018. BMC Infect Dis.

[12] Murdoch DR, Corey GR, Hoen B, Miró JM, Fowler VG, Bayer AS (2009). Clinical presentation, etiology, and outcome of infective endocarditis in the 21st century: the international collaboration on endocarditis–prospective cohort study. Arch Intern Med.

[13] Galvez-Acebal J, Almendro-Delia M, Ruiz J, de Alarcon A, Martinez-Marcos FJ, Reguera JM (2014). Influence of early surgical treatment on the prognosis of left-sided infective endocarditis: a multicenter cohort study. Mayo Clin Proc.

[14] Liang F, Song B, Liu R, Yang L, Tang H, Li Y (2016). Optimal timing for early surgery in infective endocarditis: a meta-analysis. Interact Cardiovasc Thorac Surg.

[15] Duk-Hyun K, Yong-Jin K, Sung-Han K, Byung Joo S, Dae-Hee K, Sung-Cheol Y (2012). Early surgery versus conventional treatment for infective endocarditis. N Engl J Med.

[16] Ciliberto GR, Moreo A, Lobiati E, Alberti A, Massa D, Gordini V (1999). The limitations of echocardiography in the overall diagnosis of the morphological lesions associated with infective endocarditis: comparison of echocardiographic and surgical findings. G Ital Cardiol.

[17] Strom J, Becker R, Davis R, Matsumoto M, Frishman W, Sonnenblick EH (1980). Echocardiographic and surgical correlations in bacterial endocarditis. Circulation..

[18] Perez-Garcia CN, Olmos C, Islas F, Marcos-Alberca P, Pozo E, Ferrera C (2019). Morphological characterization of vegetation by real-time three-dimensional transesophageal echocardiography in infective endocarditis: prognostic impact. Echocardiography..

[19] Incani A, Hair C, Purnell P, O’Brien DP, Cheng AC, Appelbe A (2013). Staphylococcus aureusbacteraemia: evaluation of the role of transoesophageal echocardiography in identifying clinically unsuspected endocarditis. Eur J Clin Microbiol Infect Dis.

[20] Wong D, Keynan Y, Rubinstein E (2014). Comparison between transthoracic and transesophageal echocardiography in screening for infective endocarditis in patients with staphylococcus aureusbacteremia. Eur J Clin Microbiol Infect Dis.

[21] Habib G, Derumeaux G, Avierinos JF, Casalta JP, Jamal F, Volot F (1999). Value and limitations of the Duke criteria for the diagnosis of infective endocarditis. J Am Coll Cardiol.

[22] Kanafani ZA, Kanj SS, Cabell CH, Cecchi E, Ramos ADO, Lejko-Zupanc T (2010). Revisiting the effect of referral bias on the clinical spectrum of infective endocarditis in adults. Eur J Clin Microbiol Infect Dis.

