# Supplementary material for: Preoperative false-negative transthoracic echocardiographic results in native valve infective endocarditis patients: a retrospective study from 2001 to 2018
Source: Cardiovasc Ultrasound. 2021 Jan 2;19:2. doi: 10.1186/s12947-020-00229-8 (PMC7778797; doi:10.1186/s12947-020-00229-8)
Supplement: Supplementary file 1 — Additional file 1. The anatomical and echocardiographic definitions. [file 12947_2020_229_MOESM1_ESM.docx]

| **Additional file 1. The anatomical and echocardiographic definitions*** | | |
| --- | --- | --- |
|  | **Surgery/necropsy** | **Echocardiography** |
| **Vegetation** | Infected mass attached to an endocardial structure or on implanted intracardiac material. | Oscillating or no oscillating intracardiac mass on valve or other endocardial structures, or on implanted intracardiac material |
| **Abscess** | Perivalvular cavity with necrosis and purulent material not communicating with the cardiovascular lumen. | Thickened, nonhomogeneous perivalvular area with echo dense or echo lucent appearance. |
| **Pseudoaneurysm** | Perivalvular cavity communicating with the cardiovascular lumen. | Pulsatile perivalvular echo-free space, with color-Doppler flow detected. |
| **Perforation** | Interruption of endocardial tissue continuity | Interruption of endocardial tissue continuity traversed by color-Doppler flow |
| **Fistula** | Communication between two neighboring cavities through a perforation. | Color-Doppler communication between two neighboring cavities through a perforation. |
| **Valve aneurysm** | Saccular outpouching of valvular tissue. | Saccular bulging of valvular tissue. |
| **Dehiscence of a prosthetic valve** | Dehiscence of the prosthesis. | Paravalvular regurgitation identified by TTE/TOE, with or without rocking motion of the prosthesis. |
| *Cited from the ESC guideline of IE[4]. | | |
